# Development and Evaluation of Zinc and Iron Nanoparticles Functionalized with Plant Growth-Promoting Rhizobacteria (PGPR) and Microalgae for Their Application as Bio-Nanofertilizers

**DOI:** 10.3390/plants12203657

**Published:** 2023-10-23

**Authors:** Carlos Esteban Guardiola-Márquez, Edgar R. López-Mena, M. Eugenia Segura-Jiménez, Isaac Gutierrez-Marmolejo, Manuel A. Flores-Matzumiya, Shirley Mora-Godínez, Carmen Hernández-Brenes, Daniel A. Jacobo-Velázquez

**Affiliations:** 1 Tecnologico de Monterrey, Escuela de Ingenieria y Ciencias, Campus Guadalajara, Ave. General Ramon Corona 2514, Zapopan 45138, Mexico; a01560073@tec.mx (C.E.G.-M.);; 2 Tecnologico de Monterrey, Escuela de Ingenieria y Ciencias, Campus Monterrey, Ave. Eugenio Garza Sada 2501, Monterrey 64849, Mexico; 3 Tecnologico de Monterrey, Institute for Obesity Research, Ave. General Ramon Corona 2514, Zapopan 45201, Mexico

**Keywords:** bio-nanofertilizer, iron, microalgae, micronutrients, nanoparticle, plant growth-promoting rhizobacteria, sustainable agriculture, zinc oxide

## Abstract

Micronutrient deficiencies are widespread and growing global concerns. Nanoscale nutrients present higher absorption rates and improved nutrient availability and nutrient use efficiency. Co-application of nanofertilizers (NFs) with biological agents or organic compounds increases NF biocompatibility, stability, and efficacy. This study aimed to develop and evaluate zinc and iron bio-nanofertilizers formulated with plant growth-promoting rhizobacteria (PGPR) and microalgae. Nanoparticles (NPs) were synthesized with the co-precipitation method and functionalized with *Pseudomonas* species and *Spirulina platensis* preparation. NPs were characterized and evaluated on seed germination, soil microbial growth, and early plant response under seedbed conditions. NPs corresponded to zinc oxide (ZnO; 77 nm) and maghemite (γ-Fe_2_O_3_; 68 nm). Functionalized nanoparticles showed larger sizes, around 145–233 nm. The seedling vigor index of tomato and maize was significantly increased (32.9–46.1%) by bacteria-functionalized ZnO- and γ-Fe_2_O_3_-NPs at 75 ppm. NFs at 250 and 75 ppm significantly increased bacterial growth. NFs also improved early plant growth by increasing plant height (14–44%), leaf diameter (22–47%), and fresh weight (46–119%) in broccoli and radish, which were mainly influenced by bacteria capped ZnO- and γ-Fe_2_O_3_-NPs at 250 ppm. Beneficial effects on plant growth can be attributed to the synergistic interaction of the biological components and the zinc and iron NPs in the bio-nanofertilizers.

## 1. Introduction

Micronutrient deficiencies in agricultural lands and plants are widespread and growing global concerns that negatively impact crop production and reduce the nutritional quality of food and, consequently, human health and well-being [1,2]. Intensive agriculture and overexploitation of soil resources have contributed significantly to these problems by causing the depletion of soil nutrients and water reserves [3]. Additionally, conventional mineral fertilization, now considered a crucial and unavoidable agricultural practice to provide extra nutrients to the soil, is mainly focused on applying three macronutrients (nitrogen (N), phosphorus (P), and potassium (K)) without addressing other micronutrient requirements, which has exacerbated the incidence of plant micronutrient deficiencies [4,5,6,7,8]. Insufficient quantities and low bioavailability of micronutrients in plants are in a close connection with the high prevalence of micronutrient deficiencies in humans [1,2]. Around one-third of the world’s population is affected by micronutrient deficiencies. In particular, zinc (Zn) and iron (Fe) are among the most widespread deficiencies; in fact, it is evident that 20% of deaths of children under five years old are caused by Fe and Zn deficiencies, which are strongly related to diet quality [1,2,4,6,9,10,11].

Zinc is an essential micronutrient for both plants and humans since this metal is crucial for over 300 enzymes and hormones; hence it is involved in many metabolic processes, including DNA transcription, RNA processing, protein synthesis, protein–protein interaction, carbohydrate metabolism, and cell membrane integrity. Zinc deficiency in plants is associated with physiological stress, decreased photosynthetic rate, dwarf growth, hormonal imbalances, chlorosis, spikelet sterility, decreased crop production, and reduced nutritional value of fruits and seeds [4,7,10]. In the soil, only 1% of total Zn is water-soluble and available for plant uptake (Zn^2+^); more than 84% can be found as ZnCO_3_, ZnO, Zn_3_(PO_4_)_2_·4H_2_O, and ZnS. In addition, it is estimated that approximately 50% of agricultural soil presents zinc deficiency with only 0.1–2.0 mg/kg available for plant uptake when the plant requirement for optimal production is at least 30–60 mg/kg [4]. On the other hand, iron also plays a fundamental role in enzymatic activity, nucleic acid synthesis, DNA stability/repair, electron-transfer chains, tricarboxylic acid cycle, lipid metabolism, and oxygen transport. Particularly in plants, iron has an important role in chlorophyll and phytohormone synthesis, cell respiration, and photosynthesis. Degraded croplands and alkaline soils commonly present low iron bioavailability, producing iron-deficient fruits and seeds [6,12]. Approximately 30% of the world’s lands present iron deficiency, an important problem for crops growing in that soil [13]. Therefore, there is an important need to increase vital micronutrients in agricultural products to satisfy the human nutrient requirements for normal health [4].

Promoting mineral fertilization with these micronutrients can help to reduce micronutrient deficiencies. However, the conventional application of the inorganic forms of zinc and iron implies a very low nutrient assimilation efficiency since they, when applied in the form of salt, are more soluble in water and can be lost more easily by leaching. Also, depending on the characteristics of the soil, they can bind soil particles and precipitate or fix as carbonates, hydroxides, oxides, silicates, or form complexes with other organic compounds, as in the case of calcareous soils [1,3,4,8,14,15]. Most of the soluble Zn applied in fertilizers is rapidly fixed and transformed into insoluble forms, and only 1–4% of the Zn can be used by plants [6]. Key knowledge has been developed on the production of more efficient fertilizers to supply micronutrients to plants. The use of synthetic chelators, such as HBED [N,N′-di(2-hydroxybenzyl)ethylenediamine-N,N′-diacetic acid monohydrochloride] that links Zn or Fe with high affinity and increases the availability of these metals for plant uptake is now used in the field. Chelated micronutrients for the correction of micronutrient deficiencies in plants is an expensive strategy and needs to be optimized; only 6% of micronutrient fertilizers are applied as chelates [1].

Nanofertilization has risen as an innovative and promising solution for conventional mineral fertilizers and as an important strategy to increase crop productivity, even under abiotic stress, and reduce plant nutrient deficiencies and soil nutrient imbalances [3,4,7,8,10,14,16]. Nanofertilizers (NFs) are also substances that provide nutrients to plants but in a much smaller particle size (nanoscale < 100 nm). This confers unique physicochemical properties that provide many advantages compared to the bulk mineral analogs and increase the fertilization efficiency to between 20 and 30%. Some of their characteristics include high surface area, better functionalization properties, and increased reactivity, electrical conductivity, and mechanical strength. The small size of nanofertilizers facilitates their penetration through biological barriers and their transport in the plant’s vascular system. In addition, their greater surface area results in a greater reaction surface that allows a higher number of metabolic reactions in plants, improving the assimilation, bioavailability, and function of nutrients [3,4,6,17,18]. Nutrients in the form of nanoparticles (NPs) present higher absorption rates and improved nutrient availability and nutrient use efficiency. It has been reported that the dose of nanofertilizers can be up to 100 times lower than the used for conventional mineral fertilizers, meeting more efficiently the plant nutritional requirements to promote crop growth and productivity [3,6,8,14,15,16,17]. 

Even though NFs represent a promising strategy for their positive contributions to agriculture, NFs must be carefully produced and evaluated to meet sustainability and environmental and human safety standards since some nanomaterials have presented nanotoxic effects for plants and soil microbial communities [5,19]. Depending on the NP properties (particle composition, size, morphology, solubility, surface chemistry), media composition, dose, and route of exposure, NPs can exert beneficial or toxic effects [6]. The formulation of bio-nanofertilizers (B-NFs) has emerged as an alternative to overcome nanofertilizer drawbacks; they consist of nanomaterials applied in conjunction with biological agents, such as plant growth-promoting rhizobacteria (PGPR) or organic compounds (phenolic compounds, proteins, amino acids, carbohydrates) and biostimulants that come from bacteria, fungi, microalgae, or plants and act as capping agents, enhancing NFs biocompatibility, stability, efficacy, and safety [6,11,16]. In this work, PGPR and a microalgae extract were used to produce B-NFs since, on the one hand, PGPR are beneficial microorganisms that colonize the plant’s rhizosphere and exert important processes in the soil, such as nutrient mineralization, cycling and solubilization, soil organic matter decomposition, nitrogen fixation, siderophores production, disease suppression, soil structure formation, and plant abiotic stress resistance [5,20,21]. Meanwhile, microalgae extracts, including those form *Spirulina* spp., are considered an excellent source of a wide range of beneficial compounds, such as protein, amino acids, vitamins, minerals, lipids, and polysaccharides [22,23]. 

Based on previous knowledge, the objective of the present study was to synthesize and characterize zinc and iron nanoparticles to formulate different bio-nanofertilizers based on PGPR and microalgae preparations, as well as evaluate their effects on the growth of soil microorganisms and plant seedlings to elucidate safe concentrations and identify if the nanoscale of micronutrients or the use of biological agents in the nanoformulations exert beneficial effects on the early stages of plant development.

## 2. Results

### 2.1. Synthesis and Characterization of Zinc and Iron Nanoparticles

The nanoparticle synthesis reaction was based on the co-precipitation method. Iron sulfate (FeSO_4_·7H_2_O) and ferric chloride (FeCl_3_·6H_2_O) salts were used to synthesize iron nanoparticles, while zinc acetate (ZnC_4_H_6_O_4_·2H_2_O) was used as starting material for zinc nanoparticles. A fraction of each type of synthesized nanoparticles was functionalized with biological preparations of PGPR (native *Pseudomonas* species) and microalgae biomass to incorporate a surface capping to metal NPs. Subsequently, the nanoparticles were characterized by different methods to determine their crystalline structure, size, size distribution, Z potential (mV), and surface morphology. 

#### 2.1.1. Crystal Structure and Size Conformation of the Zinc and Iron NPs 

**Figure 1:**
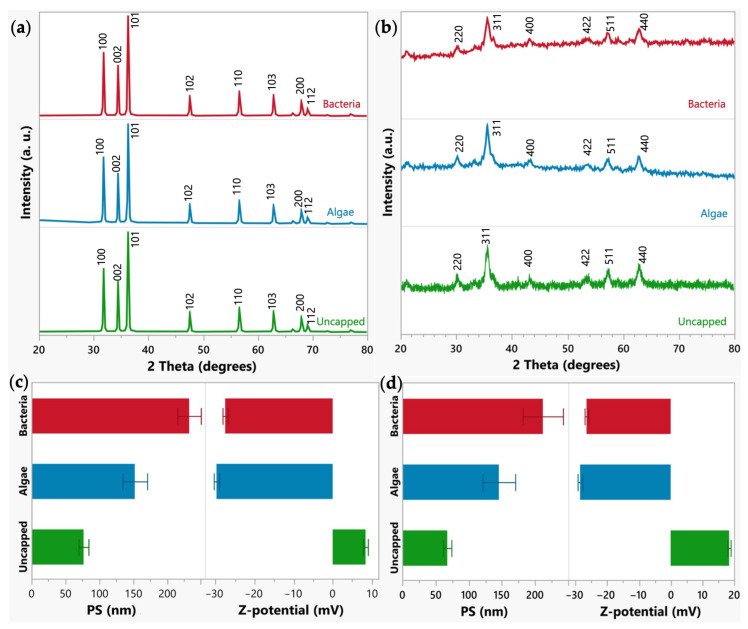
Physical characterization of the synthesized zinc and iron nanoparticles. (**a**) XRD pattern of ZnO-NPs (main crystal planes of zincite (JCPDS card no. 36-1451) are shown on the peaks). (**b**) XRD pattern of γ-Fe_2_O_3_-NPs (main crystal planes of maghemite (JCPDS card no. 39-1364) are shown on the peaks). (**c**) Particle size (PS) and Z-potential of the ZnO-NPs. (**d**) Particle size (PS) and Z-potential of the γ-Fe_2_O_3_-NPs.

The uncapped and capped zinc oxide (ZnO) XRD patterns (Figure 1a) exhibited well-defined peaks that correspond to (100), (002), (101), (102), (110), (103), (200), and (112) planes. These planes are characteristic reflections of the hexagonal wurtzite of the ZnO (JCPDF #36-1451) [24]. All the nanoparticles exhibited good crystallinity. Using the Debye–Scherrer equation, the average crystallite size of ZnO-NPs was calculated, and the results are 41.38, 39.43, and 44.22 nm (±5%) for bacteria, algae, and uncapped ZnO NPs, respectively [4]. Regarding iron nanoparticles, all the samples showed diffraction peaks related to (220), (311), (400), (422), (511), and (440) planes, which correspond to the face-centered cubic structure of maghemite (γ-Fe_2_O_3_) (JCPDS #39-1364) (Figure 1b) [25]. The average crystallite size was 16.23, 14.49, and 16.14 nm (±5%) for bacteria, algae, and uncapped γ-Fe_2_O_3_-NPs, respectively.

#### 2.1.2. Size (nm) and Zeta Potential (mV) of the NPs 

The dynamic light scattering method (DLS) was utilized to analyze the Z-potential, median hydrodynamic size, and the size distribution of the uncapped and capped ZnO and γ-Fe_2_O_3_-NPs (Figure 1c,d). The uncapped NPs showed a smaller median hydrodynamic size for both series (76.84 ± 10.3 nm and 67.7 ± 8.9 nm for ZnO- and γ-Fe_2_O_3_-NPs, respectively), attributed to the capped materials (bacteria and microalgae) promoting the NP aggregation. Bacterial treatment increased particle size to 232.85 ± 24.5 nm for ZnO-NPs and 211.73 ± 43.0 nm for γ-Fe_2_O_3_-NPs, while microalgae increased the particle size to 152.52 ± 25.8 nm for ZnO-NPs and 145.45 ± 34.9 nm for γ-Fe_2_O_3_-NPs. From the Z-potential, all the samples displayed good stability in an aqueous medium. Uncapped NPs exhibited a positive charge for ZnO-NPs (8.4 ± 0.9 mV) and γ-Fe_2_O_3_-NPs (18.42 ± 0.7 mV). The functionalization process modified the charge to negative values; bacteria capped nanoparticles showed −27.47 ± 0.8 and −26.50 ± 0.8 mV for ZnO and γ-Fe_2_O_3_, respectively, and microalgae capped presented −29.7 ± 0.9 for ZnO and −28.53 ± 1.0 mV for γ-Fe_2_O_3_. The change from negative to positive in the Z-potential may be associated with hydroxyl groups on the surface of NPs. 

#### 2.1.3. Surface Morphology and Elemental Composition of the Uncapped Zinc and Iron Nanoparticles

**Figure 2:**
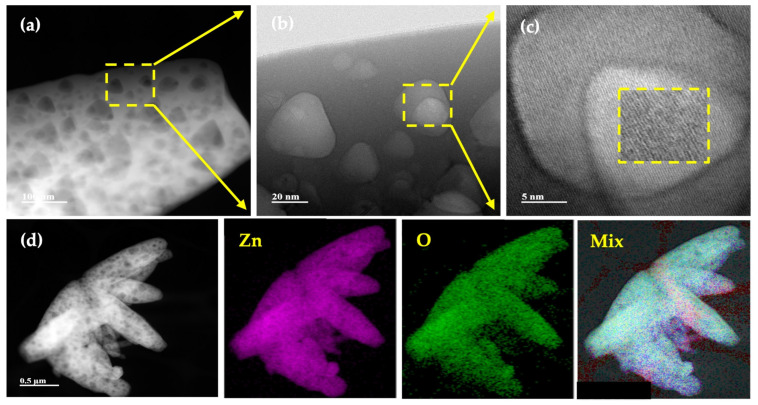
Annular dark-field (HAAF) image of ZnO-NPs at (**a**) low, (**b**) medium, and (**c**) high resolution. (**d**) Elemental mapping of Zn, O and image mix of Zn-O.

A detailed inspection of the uncapped ZnO- and γ-Fe_2_O_3_-NPs was carried out by transmission and high-resolution electron microscopy (TEM and HRTEM). Figure 2 displays the results for ZnO-NP. From these images (Figure 2a–c), it is possible to notice that the ZnO sample exhibits a leaf-like microstructure. Also, some nanoparticles are observed on the leaf’s surface. Figure 2d shows an elemental mapping and image mix of Zn and O, where it is possible to notice a homogeneous distribution of these elements.

**Figure 3:**
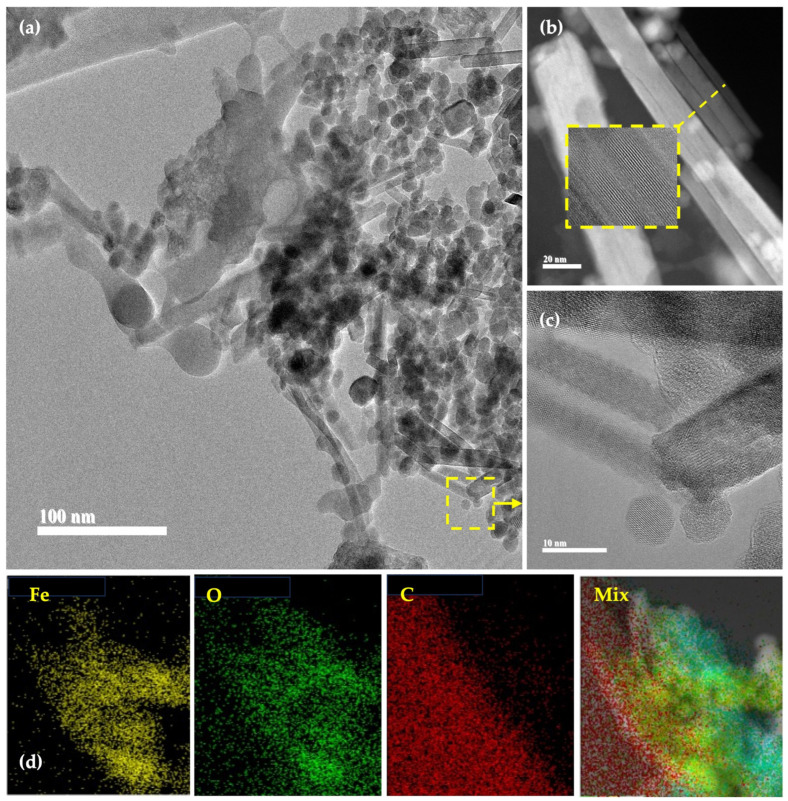
(**a**) TEM image, (**b**) Annular dark-field (HAAF) image, (**c**) HRTEM image, and (**d**) elemental mapping and mix of Fe, O, and C.

Figure 3 shows a series of transmission electron microscopy (TEM) and high-resolution transmission electron microscopy (HRTEM) images of γ-Fe_2_O_3_-NPs. Interestingly, Figure 3a shows a mixture of microstructure nanorods and nanoparticles. The nanorods and nanoparticle average diameters are 22.3 and 14.5 nm, respectively. Figure 3b (HAAF) and Figure 3c (HRTEM) show high-magnification images of the nanorods. In both images, it is possible to notice the crystalline planes of the γ-Fe_2_O_3_-NPs. Figure 3d shows the elemental mapping of Fe, oxygen (O), and carbon (C). Also, a mix of elemental mapping is shown. 

### 2.2. Evaluation of the Plant and Microbial Growth Promotion/Inhibition Effects of the Zinc and Iron Nanoformulations 

Synthesized capped and uncapped nanoparticles were evaluated on the germination of tomato and maize, the growth of native soil microorganisms, and the early plant response of broccoli and radish sprouts to determine safe/harmful concentrations and recognize potential nanoformulations for their application as nanofertilizers. Once nanotoxicity effects were identified in the first tests, the concentrations responsible for such effects were discarded for the subsequent studies. The treatments considered in each assay are listed in Table 1. 

#### 2.2.1. Effect of Zinc and Iron Bio-Nanoformulations on the Germination of Two Relevant Food-Crops

The impact of bio-nanoformulations was evaluated on the germination of tomatoes and maize. Twenty-five seeds per treatment were tested in tomato, and ten seeds per treatment in maize. The number of germinated seeds was measured to determine the germination percentage and root and shoot length to estimate the vigor index. 

**Figure 4:**
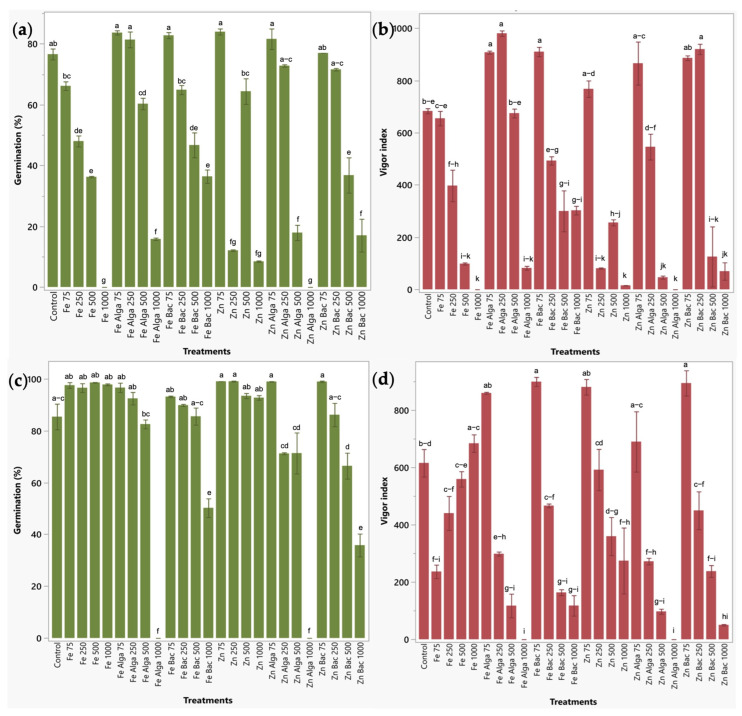
Evaluation of bio-nanoformulations on the germination of tomato and maize. (**a**) Germination (%) of tomato. (**b**) Vigor index of tomato seeds. (**c**) Germination (%) of maize. (**d**) Vigor index of maize. Results are shown as mean ± standard deviation. Letters above the bars indicate significant statistical differences between treatments (*p* < 0.05).

Germination of tomato seeds was significantly reduced by all the treatments applied at 500 and 1000 ppm (15.93–100%). Also, the uncapped ZnO and γ-Fe_2_O_3_-NPs at 250 ppm significantly diminish the germination percentage by 84.25% and 37.34%, respectively (Figure 4a). With respect to the vigor index of tomato plants, it was significantly increased between 34.66 and 43.54% by using γ-Fe_2_O_3_-NPs functionalized with microalgae and bacteria-treated ZnO-NPs applied at 250 ppm, and between 32.89 and 33.23% with microalgae capped γ-Fe_2_O_3_-NPs and bacteria treated ZnO-NPs at 75 ppm. The same parameter was significantly decreased by all the treatments applied at 500 and 1000 ppm (except for microalgae functionalized γ-Fe_2_O_3_-NPs at 500 ppm) (Figure 4b). 

Regarding maize seeds, germination was significantly reduced by nearly all the treatments at 1000 ppm (41.18–100%) (except for uncapped NPs) and by microalgae-treated ZnO-NPs at 500 ppm (22.20%) (Figure 4c). The vigor index was improved in this crop by using bacteria-functionalized ZnO- and γ-Fe_2_O_3_-NPs applied at 75 ppm, which increased this parameter between 45.30 and 46.12%. The vigor index was also diminished by most of the nanoformulations at 500 and 1000 ppm, attributing nanotoxic effects to these treatments (Figure 4d).

#### 2.2.2. Impact of Bio-Nanoformulations on the Growth of Soil Microorganisms

Microbial growth was evaluated as a starting point to identify safe concentrations of bio-nanoformulations for soil environments. Uncapped and capped zinc and iron nanoformulations were added at three concentrations (75, 250, 500 ppm) to a pre-culture of *P. marginalis* in the TSB medium. Initial and final bacterial concentrations were estimated (Table 2).

Based on the results, *Pseudomonas* growth was significantly reduced by uncapped γ-Fe_2_O_3_-NPs and γ-Fe_2_O_3_-NPs capped with microalgae extract at 250 ppm and by all the treatments at 500 ppm. In the rest of the treatments at 250 and 75 ppm, the increase in bacterial biomass was significantly higher than the control, except for uncapped γ-Fe_2_O_3_-NPs at 75 ppm. Treatments based on nanoparticles functionalized with bacteria were tested to check bacterial cell viability after the functionalization process and after one month of storage. In both cases, bacteria remained viable with an average cell density of 1.2–1.4 × 10^8^ colony-forming units per mL (CFU/mL).

The effect of bio-nanoformulations was also evaluated on fungal growth by measuring the impact on the fungal radial growth. The *Penicillium* strain did not show visual changes in its growth with any of the treatments. It was not possible to measure radial growth since, after the incubation period, it was distributed over the entire surface of the medium. Regarding the growth of *Fusarium*, only uncapped γ-Fe_2_O_3_-NPs at 500 ppm and γ-Fe_2_O_3_-NPs treated with bacteria at 500 ppm exerted a significant impact on radial growth, reducing it between 8.89 and 10% with respect to the control.

#### 2.2.3. Early Plant Response to Bio-Nanoformulations in a Seedbed Assay

Zinc and iron bio-nanoformulations were evaluated in a 12-day seedbed assay in radish (*Raphanus sativus* L. var. ‘Champion’) and broccoli (*Brassica oleracea* L. var. *italica*) to identify their impact on the first stages of plant development. Plant height, leaf diameter, root length, and shoot fresh weight were measured to evaluate the nanofertilizer effect.

**Figure 5:**
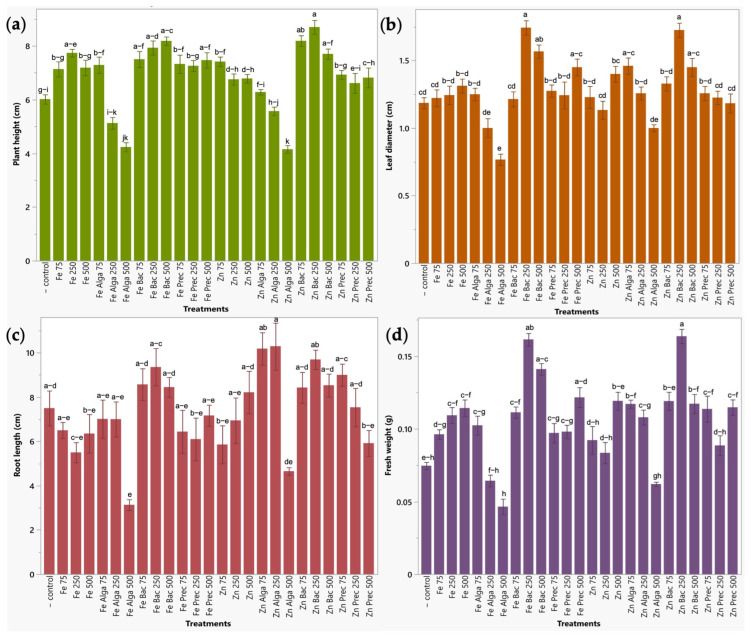
Evaluation of zinc and iron bio-nanoformulations on agronomic parameters in broccoli sprouts. (**a**) Plant height (cm). (**b**) Leaf diameter (cm). (**c**) Root length (cm). (**d**) Fresh weight (g). Results are expressed as mean ± standard deviation, and letters above the bars indicate significant differences between treatments (*p* < 0.05).

Regarding broccoli sprouts, plant height was significantly increased by ZnO-NPs treated with bacteria at all concentrations tested (500, 250 and 75 ppm) (Zn Bac 500, Zn Bac 250, Zn Bac 75), γ-Fe_2_O_3_-NPs functionalized with bacteria also at all concentrations (Fe Bac 500, Fe Bac 250, Fe Bac 75), uncapped γ-Fe_2_O_3_-NPs at 250 ppm (Fe 250) and mineral precursor of iron nanoparticles at 500 ppm (Fe Prec 500). Zinc treatments significantly improved (*p* < 0.05) plant height between 28.03 and 44.66%, while iron treatments between 24.15 and 36.06% as compared with untreated plants. Leaf diameter showed significant increases between 22.29 to 45.48% when treated with zinc bio-nanoformulations (bacteria treated ZnO-NPs at 250 and 500 ppm (Zn Bac 250, Zn Bac 500), and microalgae treated ZnO-NPs at 75 ppm (Zn Alga 75)), and between 22.29 to 46.99% when using iron nanoparticles (250 and 500 ppm) capped with bacteria (Fe Bac 250, Fe Bac 500) and ferric and ferrous salts at 500 ppm (Fe Prec 500) compared to water-irrigated plants. Measurements of root length did not lead to statistically significant results. This metric exhibited high variability, probably due to changes in the development of both primary and lateral roots, likely attributed to the dimensions of the seedbed cavities. Root structures were tangled and were handled carefully to prevent fracture and consequent loss of plant material, which could impact weighing procedures. Finally, fresh weight was significantly improved up to 119.30% by using ZnO-NPs functionalized with bacteria at 250 ppm. Iron treatments (Fe Bac 250, Fe Bac 500, Fe Prec 500) also increased fresh weight from 63.14 to 116.35% with respect to water-irrigated plants (Figure 5). 

**Figure 6:**
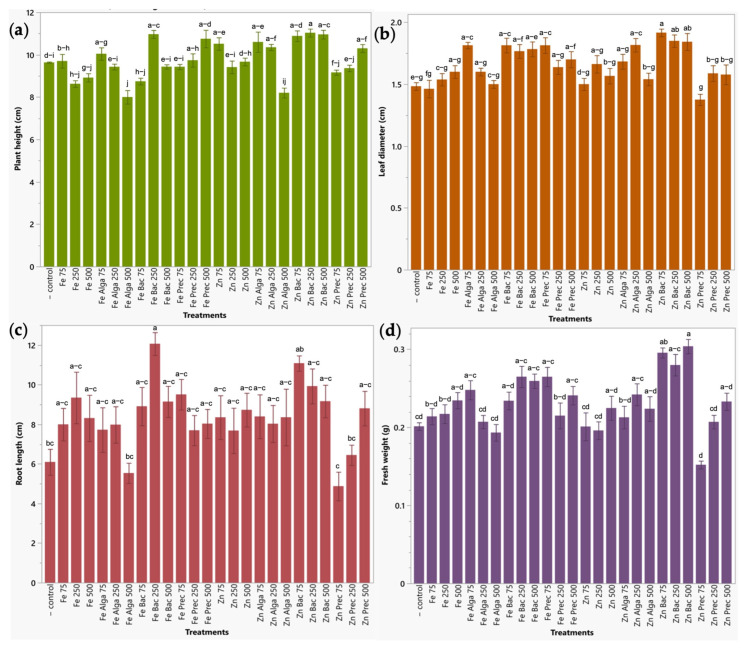
Evaluation of zinc and iron bio-nanoformulations on agronomic parameters in radish sprouts. (**a**) Plant height (cm). (**b**) Leaf diameter (cm). (**c**) Root length (cm). (**d**) Fresh weight (g). Results are expressed as mean ± standard deviation. Letters above the bars indicate significant differences between treatments (*p* < 0.05).

Radish exhibited similar results to those obtained in broccoli. Plant height of radish was significantly improved (13.74–14.53%) by bacteria-capped ZnO-NPs at 500 and 250 ppm (Zn Bac 250 and Zn Bac 500) and by γ-Fe_2_O_3_-NPs also treated with bacteria at 250 ppm (Fe Bac 250) with a 13.84% increase. Leaf diameter presented significant increases between 22.48 to 29.22% by using zinc bio-nanoformulations (bacteria treated ZnO-NPs at all concentrations tested (500, 250 and 75 ppm) (Zn Bac 500, Zn Bac 250, Zn Bac 75), and microalgae treated ZnO-NPs at 250 ppm (Zn Alga 250)), and between 22.18 to 22.32% in plants treated with bacteria and microalgae capped iron nanoparticles and with the mineral precursor of iron nanoparticles, all at 75 ppm, as compared to water-irrigated plants. As observed in broccoli sprouts, the root length parameter did not lead to conclusive results. Finally, Zn bac 500 and Zn bac 75 treatments (bacteria-functionalized ZnO-NPs at 500 and 75 ppm) were the only ones that significantly increased fresh weight (46.77–50.90%) compared to the negative control (Figure 6).

## 3. Discussion

In recent years, the use of nanoparticles as nanofertilizers has been increasingly studied due to their benefits in nutrition management. Nanofertilizers possess both the physicochemical characteristics of a nanomaterial and the properties of a nutrient; they have emerged as a promising alternative to conventional mineral fertilizers because of their increased nutrient use efficiency [15,26,27]. However, implementing this technology in the agricultural market has been limited due to uncertainties regarding their eco-toxicity, which has limited large-scale use [5,27]. Previous reports have indicated that the beneficial effect or toxicity of nanoparticles on agroecosystems depends mainly on their size, chemical composition, media composition, dose, solubility, route of exposure, and soil status [5,28,29]. Therefore, in this study, a simple, industrially viable, and economical method to synthesize nanoparticles was employed, which can be standardized by adjusting the pH, temperature, reducing agents, and solvents to obtain reproducible results regarding the characteristics of the nanoparticles [30]. 

Safety assessment methods are required to evaluate the effect of nanofertilizers on agroecosystems, including their interactions with plants and microorganisms [5]. Greater investigation of the biological reactivity of NPs must be carried out to overcome safety concerns. Saraiva et al. [3] listed different guiding principles to be considered for the safe application of nanofertilizers, and some of them include selecting materials that have already been studied and used due to their safety (high biocompatibility and biodegradability and reduced cytotoxicity), such as those used in the medical and pharmaceutical field, packaging materials and sensors, or those produced by green synthesis; applying the nanofertilizer in the specific crops in which it has been previously evaluated; including biostimulant organic substances in nanofertilizer formulations; and promoting the development and use of commercial nanofertilizers to constitute them as a viable and reliable option for producers to increase crop yields, improve soil conditions and reduce the consumption of conventional chemical fertilizers [7]. Thus, in this work, a functionalization process was implemented using different biological preparations (bacteria and microalgae) to incorporate biocomponents that can act as organic ligands to increase the stability, biocompatibility, and effectiveness of nanomaterials, which has been recommended when the nanoparticles are produced for their application in agriculture [6]. The biological preparations were carefully selected to use agents commonly applied as biofertilizers and combine them with potential nanofertilizers, thus obtaining the benefits of both in a single bio-nanofertilizer. 

Seed germination tests of agri-food crops and seedbed assays revealed that the bio-nanoformulations (nanoparticles treated with biological preparations) exerted low toxicity and significantly greater plant growth promotion effects as compared with the same nanoparticles without the functionalization process (uncapped nanoparticles). Other authors have reported similar results of this type of “green nanoparticles” in comparison with chemically or physically synthesized nanoparticles alone [31,32]. Production processes should be extensively examined to establish protocols capable of generating environmentally and biologically safe nanomaterials [14].

The effect of nanofertilizers is highly dependent on the dose. In the seed germination tests, it was found that most of the toxic effects were identified in treatments applied at a concentration of 500 ppm and, particularly, 1000 ppm; therefore, the latter concentration was removed from subsequent trials. Nanotoxicity may induce oxidative stress due to an imbalance between the production of reactive oxygen species (ROS) and the plant defense system, which can generate DNA and cell membrane damage, protein oxidation, lipid peroxidation, electrolyte leakage, and cell death [6]. The treatments with the best effects in the germination stage, considering both the germination percentage and the vigor index in tomato and maize for zinc and iron, were capped ZnO- and γ-Fe_2_O_3_-NPs applied at 75 ppm. Moderate doses of NFs were also recommended in other studies. Singh et al. [32] indicated that green ZnO-NPs (35 nm) applied at 62 ppm showed the most significant improvement in wheat seed germination and seedling growth relative to other concentration levels. Asmat-Campos et al. [33] also revealed that concentrations of ZnO-NPs (30 nm) close to 100 ppm (63.59 and 99.076 ppm) are the best to enhance tomato seeds germination since they promote metabolic activity to induce cell elongation, no phytotoxicity was observed. Itrouthwar et al. [34] also evaluated ZnO-NPs (37 nm) in maize; they found that the concentration of 100 ppm exhibited the highest seed germination rate and seedling growth parameters as compared with zinc acetate (ionic control) and water (normal control). Regarding iron oxide nanoparticles, γ-Fe_2_O_3_ NPs used at low concentrations were also observed to exert beneficial effects on plant growth and seed germination [35,36]. Montoya-Giraldo et al. [37] reported that no phytotoxic effects from the application of aqueous suspensions of iron oxide nanoparticles at a moderate dose of 50 ppm were found on the in vitro germination and growth of maize and lettuce seeds. 

Seed treatment with zinc and iron nanoparticles and PGPR can trigger several metabolic processes in the seeds and enhance the germination rates and, particularly, the seedling vigor index. First, zinc and iron nanoparticles can improve germination and seedling development by improving the seed water uptake; NPs interact with the cell walls of the seed coat and create nanopores that increase water uptake and the expression of aquaporin genes [38,39]. That is, nanoparticles increase the penetrability of the seed and ease the entrance of water and oxygen into the cells, accelerating seed germination [40]. Also, zinc oxide nanoparticles are reported to increase the α-amylase activity up to 45%, and this hydrolytic enzyme is involved in the degradation of seed reserve carbohydrates into soluble sugars that are required to generate energy and maintain respiratory metabolism for germination and seedling growth until growth can be sustained by photosynthesis. Enhanced α-amylase activity is correlated with the increase in seed water uptake that generates higher metabolic activity [38,39]. On the other hand, Fe plays an important role in respiration (electron transport), DNA synthesis, and photosynthesis, which are crucial in the early stages of germination and seedling expansion. Both zinc and iron are cofactors or metal components of many enzymes [39,41]. An increased seedling vigor index can also be attributed to the role of micronutrients in the biosynthesis of phytohormones, such as auxins and gibberellins, and the metabolism of carbohydrates and proteins that improve emergence and seedling growth [38]. PGPR can also enhance germination by producing growth hormones, such as indole-3-acetic acid (IAA) and gibberellic acid (GA), solubilizing minerals, and releasing enzymes and biostimulants substances that promote plant development [42,43].

Even when Zn and Fe NFs have been widely studied and have revealed important effects to improve plant growth and nutrition, zinc and iron nanoparticles have also been frequently investigated for their antifungal and antibacterial properties [44,45,46,47,48,49]. Therefore, when nanomaterials are developed for their application in agriculture, it is crucial to evaluate their impact on soil microorganisms [14]. Soil microbiota plays a fundamental role in agriculture, contributing to several aspects of soil health and fertility, plant growth, nutrient cycling, and agricultural productivity. Beneficial microorganisms colonize the plant roots, establish symbiotic relationships with plants, and benefit plant development by enhancing plant nutrient uptake and assimilation and plant resistance against various biotic and abiotic stresses [20]. In this study, the effect of nanoformulations on the growth of different microbial strains was evaluated; both bacterial and fungal species were mainly affected by treatments applied at 500 ppm. However, fungal species were much less affected by nanoparticles compared to bacteria. Interestingly, most of the treatments applied at 250 and 75 ppm significantly enhanced bacterial growth; it is of great importance to identify nanofertilizers that, in addition to improving plant growth, are also capable of inducing beneficial effects on soil microbial populations [16]. Verma et al. [8] reported that nanofertilizers can improve soil fertility by stimulating microbial communities. Wei et al. [50] evaluated the impact of a 165-day exposure of ZnO (20 nm) and γ-Fe_2_O_3_ (50 nm) nanoparticles on the rhizosphere microbial communities and underground biomass of the medicinal plant *Salvia miltiorrhiza* (Bge.). They found that ZnO and γ-Fe_2_O_3_ NPs significantly improved plant growth by increasing plant root diameter and underground biomass and increased the relative abundance of beneficial bacterial genera (*Humicola*, *Arenimonas*, *Thiobacillus,* and *Methylobacillus*) between 97.46% and 297.14%. Zinc and iron nanoparticles can serve as a source of nutrients for microorganisms, and these micronutrients also exert vital functions for microbial growth and metabolism, including enzyme cofactor, protein synthesis, regulation of gene expression by modulating the activity of transcription factors, membrane stability by interacting with membrane proteins and lipids, DNA stability and repair, energy metabolism and electron transfer, oxidative stress resistance, among others [51]. Zinc and iron ions can slowly be released from the nanoparticles for microbial utilization. Bacterial isolates used in the treatment of nanoparticles were previously evaluated for several plant growth-promoting traits, exhibiting potential zinc solubilizing activity and siderophores production [20]. Microorganisms solubilize zinc by producing different types of organic acids (citric, lactic, acetic, and gluconic acids) to reduce the pH of the environment; an acidic medium enhances the dissolution of nanoparticles. Microbial siderophores can scavenge Zn^2+^ and Fe^3+^ from the nanoparticles and form soluble Zn^2+^- and Fe^3+^-siderophores complexes [13,20,52].

In addition to the effect of NP concentration, an important distinguishing property of nanofertilizers is their size. This study evaluated particle sizes around 76.84 ± 10.3 nm and 67.7 ± 8.9 nm for uncapped ZnO- and γ-Fe_2_O_3_-NPs, respectively; functionalized nanoparticles had even larger sizes, which may be related to the reduced toxicity of these nanomaterials since it is described that NPs with smaller size frequently show higher toxicity to the plants compared to larger NPs [53]. For example, nanoparticles of less than 5 nm are reported to induce phytotoxicity even at low concentrations [54]. Small-sized NPs, due to their increased surface-to-volume ratio, have higher reactivity and interact more extensively with the plant tissues and the surrounding environment. Small NPs are easily transported within the plant tissues and may have higher levels of accumulation and toxicity, and they are more likely to generate oxidative stress within plant cells [54,55].

Regarding the effect of bio-nanoformulations on the first stages of plant development, both crops were significantly improved by zinc and iron treatments. All the agronomic parameters were significantly improved, mainly by ZnO- and γ-Fe_2_O_3_-NPs treated with bacteria at 250 and 500 ppm, as compared with uncapped nanoparticles, NP precursors, and control. This response can be a result of the dual action of both components in these bio-nanoformulations: (1) the biofertilizer effect generated by the PGPR (native *Pseudomonas* species) that can provide beneficial molecules to the plants such as phenolic compounds, enzymes, phytohormones, biostimulants and organic acids, and promote their growth through different plant growth promoting traits including mineral solubilization, ammonia production, nitrogen fixation, siderophores secretion, among others [20]. And (2) the nanofertilizer function of the ZnO- and γ-Fe_2_O_3_-NPs, which provide more bioavailable essential nutrients with vital functions in the plant cells. The organic and inorganic components of these treatments could also be responsible for microbial growth promotion [10,12]. Plants can either absorb zinc and iron in the form of ions (Zn^2+^ and Fe^3+^), siderophore complexes, or NPs through the roots. Zinc or iron ions (Zn^2+^, Fe^2+,^ or Fe^3+^) can be released from the NPs into the soil, a process that can be favored by the action of PGPR and contact with root exudates [13]. Regarding iron solubilization and acquisition, plants can acidify the soil by the action of the H-ATPase AHA2, which extrudes protons and can dissolve iron through cation exchange, ferric (Fe^3+^) is reduced to ferrous (Fe^2+^) by ferric chelate reductase (FRO2) and Fe^2+^ can be then absorbed by the root cells through the metal carrier IRT1 (plasma membrane-localized Fe^2+^ transporter) [56]. Additionally, some plants can secrete phytosiderophores that can scavenge Fe^3+^ from the nanoparticles and other insoluble iron sources, and the phytosiderophore transporters secrete substances, such as mugineic acid. Fe^3+^ is subsequently chelated and forms soluble Fe^3+^-siderophores complexes that can be taken up by plants through the Yellow Stripe1-Like (YSL) transporters [13,20,52,56]. However, this mechanism of iron chelation through phytosiderophores is primarily used by grasses. Non-Gramineae plants neither produce nor efficiently use phytosiderophores, but as mentioned above, the bacterial isolates applied with the nanoparticles are capable of producing siderophores and perform the same function as phytosiderophores [56]. Similarly, zinc ions (Zn^2+^) can be released from the NPs into the soil by the acidification of the rhizosphere; this process is mediated by root exudates but also the release of organic acids such as (gluconic, citric, lactic, and acetic acids) by soil microorganisms [13,56]. In this sense, the bacteria used in this work exhibited important zinc solubilizing activity in previous assays, which is achieved by the production of those organic acids that reduce the pH in the rhizosphere [20]. Zn is then absorbed by root epidermal cells through transmembrane transporters of the ZIP (ZRT and IRT-like protein) family [56]. Zinc can also be absorbed through the siderophore mechanism by forming Zn^2+^-siderophores complexes. Nanoparticles can also be absorbed by the roots, and their biochemical transformations occur inside the plant tissues [13,20].

It is important to mention that bulk materials (iron sulfate (FeSO_4_·7H_2_O) and ferric chloride (FeCl_3_·6H_2_O) salts) that were used to synthesize iron nanoparticles also improved agronomic parameters when applied at 500 ppm; however, only this concentration achieved the same effects as nanoparticles applied at 250 ppm to improve plant development; a higher concentration of bulk materials of both micronutrients is required to achieve the desired effects on plants [18,29]. In general, the application of NPs exerted a greater impact on microbe and plant growth than bulk mineral salts, which can be attributed to the better absorption and assimilation of the NPs by plants due to their nanoscale and lower solubility; there is a more gradual and slower release of zinc and iron ions contained in the NPs, compared to the bulk minerals that present high water solubility and very low retention within the plant [38]. Nanofertilizer advantages over various forms of mineral fertilizers currently in use are reported in several studies [4,5,11]. This can be an initial finding to start studying the potential of these nanomaterials to correct micronutrient deficiencies. Verma et al. [8] reported that zinc nanofertilizer is required 10 times less than standard ZnSO_4_. Nanofertilizers can improve nutrient absorption and utilization, reduce leaching of nutrients, and prevent their volatilization into the atmosphere [14].

Soil properties also influence the absorption of zinc and iron [56]. In the present work, black soil with neutral pH was used for the seedbed assay. This substrate was used to obtain a response closer to what happens in the field, where the applied products interact with the soil components and can vary their performance. It is reported that soil pH is one of the most important factors determining the availability of plant nutrients. In the case of micronutrients, their availability commonly decreases as pH increases; as the pH increases, the solubility of free Fe can decrease up to 1000-fold. A soil with a neutral to alkaline pH with high clay, carbonate, and silica contents, like the one used in this study, causes a decrease in the availability of zinc and iron [20,56]. Even when there is zinc and iron present in the soil, they may not be available for plant uptake. As mentioned above, for zinc, only 1% is water-soluble (Zn^2+^), and more than 84% can be found fixed or converted to insoluble forms [4]. Therefore, the mechanisms described above regarding plant and microbial micronutrient solubilization and chelation play a very important role in increasing the bioavailability, absorption, and assimilation of zinc and iron. Nevertheless, in the field, conditions such as temperature and humidity changes and abiotic stress can negatively affect the root activity and reduce the production of root exudates [56]. Thus, finding and evaluating potential products that combine nanoscale nutrients and PGPR may constitute an important strategy to provide an important micronutrient source and increase micronutrient bioavailability by the microbial mineral solubilization and siderophores production activities, improving plant nutrition and helping plants overcome adverse conditions.

## 4. Materials and Methods

### 4.1. Chemicals and Plant Material

Iron (II) sulfate heptahydrate (FeSO_4_·7H_2_O) and ferric chloride (FeCl_3_·6H_2_O) were purchased from J.T. Baker Chemical Co. (Phillipsburg, NJ, USA). Zinc acetate dihydrate (ZnC_4_H_6_O_4_·2H_2_O) and ammonium hydroxide (NH_4_OH) were obtained from Sigma-Aldrich (Saint Louis, MO, USA). Dried microalgae biomass (*Spirulina platensis*) was provided by the BIOMEX^®^ company (Guadalajara, Mexico). Maize (*Zea mays* L. var ‘Ocelote’) and tomato (*Solanum lycopersicum* L. var ‘PAIPAI F1’) seeds were obtained from ASGROW^®^ (Tlajomulco, Mexico) and VitaliS^®^ (Salinas, CA, USA) companies, respectively, while radish (*Raphanus sativus* L. var. ‘Champion’) and broccoli (*Brassica oleracea* L. var. *italica*) commercial seeds Vita^®^ were from the ‘Rancho Los Molinos’ company (Tepoztlan, Mexico). 

### 4.2. Synthesis of Zinc and Iron Nanoparticles

A simple co-precipitation method was adopted to synthesize zinc and iron nanoparticles. Precursors for the nanoparticles were iron (II) sulfate heptahydrate (FeSO_4_·7H_2_O), ferric chloride (FeCl_3_·6H_2_O), and zinc acetate dihydrate (ZnC_4_H_6_O_4_·2H_2_O), the solutions of each precursor were prepared in deionized water (Milli-Q) [57,58,59,60,61]. To synthesize iron nanoparticles, Ferric and Ferrous salts were used as the starting materials, and a mixture of both salts was prepared by using 0.6 M FeCl_3_ and 0.3 M FeSO_4_ (Molar ratio of Fe^3+^:Fe^2+^ = 2:1) solution [62,63]. A 0.5 M solution of zinc acetate was used as a precursor solution for zinc nanoparticles. Briefly, 500 mL of a solution of each precursor was constantly stirred at 70 °C for 30 min using a magnetic stirrer. Simultaneously, a 30% ammonium hydroxide (NH_4_OH) solution was prepared in 100 mL of distilled water and stirred at 30 °C for 10 min. Later, ammonium hydroxide was added to precursor solutions dropwise and continuously stirred (400 rpm) at a constant reaction temperature of 70 °C for 3 h. A highly alkaline pH (9 to 14) medium was maintained. After the reaction time, the transparent zinc solution became white milky, while the iron solution changed from yellow to black. The obtained solution was allowed to settle for 24 h. The supernatant (ammonium hydroxide solution) was discarded, and nanoparticles were centrifuged (4000 rpm for 10 min) and washed five times using deionized water to remove the remaining ammonium hydroxide. Zinc-NPs were dried in an oven at 60 °C for 24 h and then calcined at 300 °C for 3 h in a muffle furnace. Iron samples were only dried at 60 °C for 24 h. Obtained samples were ground using mortar and pestle and stored in an airtight container at room temperature for further analysis and preparations [64,65].

### 4.3. Functionalization of Nanoparticles with Organic Substrates

To incorporate a surface capping on zinc and iron nanoparticles, NPs were functionalized with biological preparations corresponding to (1) a bacterial consortium of native *Pseudomonas* species that were previously isolated, characterized for several plant growth-promoting (PGP) traits, and identified through DNA sequencing [20], and (2) dried microalgae biomass (*Spirulina platensis*). 

#### 4.3.1. Preparation of Biological Substrates

Four *Pseudomonas* species (*Pseudomonas allii* strain B5KEA, *Pseudomonas marginalis* strain B9M, *Pseudomonas protegens* strain A276, *Pseudomonas sesami* strain A137A1) were individually inoculated in 500 mL flask containing 250 mL of trypticase soy broth (TSB) medium and incubated on a rotary shaker at 160 rpm and 30 °C for 48 h. Bacterial growth was monitored spectroscopically by measuring optical density at 600 nm (OD_600_) with a microplate reader VARIOSKAN LUX (ThermoFisher Scientific, Waltham, MA, USA). Cultures with OD_600_ between 0.6 and 0.8 were used, which corresponded to plate counts of 1.2 × 10^8^–1.6 × 10^8^ CFU mL^−1^ [20]. 

The commercial cultivation system for *Spirulina platensis* consisted of open raceway ponds built inside greenhouses without extra temperature and light control. Standard Zarrouk culture medium (NaHCO_3_ 16.8 g/L; NaNO_3_ 2.5 g/L; K_2_HPO_4_ 0.5 g/L; K_2_SO_4_ 1.0 g/L; NaCl 1.0 g/L; MgSO_4_·7H_2_O 0.2 g/L; CaCl_2_ 0.04 g/L; FeSO_4_·7H_2_O 0.01 g/L; EDTA 0.08 g/L and micronutrient solution) was used for microalgae growth. Microalgae biomass was recovered from the liquid culture by filtration and dried at room temperature, protected from direct sunlight. The provided microalgae powder was kept at −20 °C until use. To prepare the extract for NP functionalization, 12.5 g of *S. platensis* biomass were suspended in 500 mL of distilled water and were subjected to mechanical dispersion using a T 25 digital ULTRA-TURRAX^®^ (IKA Works, Inc., Wilmington, NC, USA) running at 24,000 rpm for 45 s, and the processing cycle was repeated twice [66,67]. Homogenized solution was then sonicated in an ultrasonic water bath at maximum power output and a frequency of 40 kHz for 15 min to ensure cell wall lysis and proper extraction of intracellular contents [59,66,68,69,70].

#### 4.3.2. Surface Capping of Zinc and Iron Nanoparticles

One gram of nanoparticles was re-dispersed in 500 mL of deionized water (Milli-Q) and bath sonicated for 10 min. The obtained dispersion was added with 500 mL of each biological preparation (bacteria and microalgae individually) [71,72]. Reactions were maintained for 8 h at 30 °C under constant stirring at 150 rpm. At the end of the reaction, nanoparticles were kept in solution at 4 °C for their characterization and evaluation in plants. A fraction of the nanoparticle suspension was centrifuged at 4500 rpm for 15 min. The NPs were dried at 60 °C for 24 h before characterization [73].

### 4.4. Characterization of Zinc and Iron Nanoparticles

Size, crystalline structure, size distribution, Z potential (mV), and surface morphology of the NPs were assessed with X-ray diffraction (XRD), Dynamic Light Scattering (DLS), and Transmission Electron Microscopy (TEM) analysis. 

#### 4.4.1. X-ray Diffraction (XRD)

X-ray diffraction analysis was performed to study the crystal structure and size conformation of the NPs [74,75,76]. Nanoparticle powders were characterized using a D-8 Advance diffractometer (Burker, Billerica, MA, USA), rotation activated, *Cu* anode λ = 1.5406 Å. XRD patterns were acquired from a 20° to 80° (2θ) with a 0.01° step size [44,74,75,76].

#### 4.4.2. Dynamic Light Scattering (DLS)

Dynamic Light Scattering (DLS) measurements were acquired using the Zetasizer Nano Series (Malvern Panalytical, Malvern, UK) to estimate the size (nm) and zeta potential (mV) of the nanoparticles. Nanoparticle suspensions (250 ppm) were filtered using a 0.22 µm membrane filter and bath sonicated for 5 min before the measurements [12,75,77]. 

#### 4.4.3. Electron Microscopy 

A high-resolution transmission electron microscope (HR-TEM, JEM-2200FE+Cs, JEOL, Tokyo, Japan) coupled with energy-dispersive X-ray spectroscopy (EDS) was employed to investigate the particle size, surface morphology, and elemental composition of the uncapped zinc and iron nanoparticles Samples were suspended in isopropyl alcohol, deposited on carbon-coated TEM grids and dried by incubation at 37 °C [12,74,75,77].

### 4.5. In Vivo Evaluation of Zinc and Iron Nanoformulations 

The effect of uncapped and capped nanoparticles was evaluated in (1) tomato and maize germination, (2) native soil microbial growth, and (3) broccoli and radish seedling growth. To identify safe concentrations or nanotoxic effects, six nanoformulations resulting from three capping conditions (uncapped or capped with bacteria or microalgae) and two micronutrients (iron and zinc), two metal precursors (iron sulfate and zinc acetate), and four concentrations (75, 250, 500, 1000 μg mL^−1^) were assessed with three replicates. To prepare nanoparticle suspensions, solutions were diluted with deionized water and homogenized with vortex for 3 min. The pH of each suspension was adjusted to 6.7 with hydrochloric acid (HCl) and 0.1 N sodium hydroxide (NaOH) before the applications [78].

#### 4.5.1. Effects on Seed Germination of Relevant Food Crops

A seed germination test was performed on maize (*Zea mays* L. var ‘Ocelote’) and tomato (*Solanum lycopersicum* L. var ‘PAIPAI F1’) seeds. These crops were selected to measure the impact of bio-nanofertilizers on the first stages of plant development in two relevant annual food-crops. Seeds were surface sterilized by using 70% ethanol for 1 min, followed by 10% sodium hypochlorite solution for three min and five washes with sterile distilled water [79]. Seeds were transferred to Petri dishes with two sheets of sterile and wetted filter paper. Plates were incubated in growth chambers at 25 °C and 70% relative humidity under a 16 h:8 h light:dark cycle. Twenty-five and ten seeds per treatment were used for tomato and maize, respectively. Three milliliters of each treatment solution were applied to the Petri dishes. Each plate was treated as one replicate, and three replicates were considered. Controls were seeds treated with sterile deionized water. Two milliliters of distilled water were added on alternate days to all plates to prevent drying. The number of germinated seeds was monitored and counted on days 12, 15, 18, 21, and 24 for maize and tomato to determine the final germination percentage, and seeds were counted as germinated when their radicle grew at least 2 mm in length [80,81,82,83,84]. The experiment was repeated twice, one month apart. At the end of the assay, root and shoot length were measured to estimate the vigor index, which was calculated as described by Win et al. [81] using the following equation:

Vigor index = Germination% × seedling length (root + shoot length) (1)

#### 4.5.2. Effects on the Growth of Soil Microorganisms

*Pseudomonas marginalis* was studied for its sensitivity/growth in the presence of various concentrations of Zn- and Fe-NPs. A single colony was grown in 5 mL of TSB medium and incubated on a rotary shaker at 180 rpm and 30 °C for 48 h. Optical density was measured at 600 nm (OD_600_) to estimate colony-forming units per mL (CFU/mL) using a conversion equivalence of 0.5 OD_600_ corresponding to 1 × 10^8^ CFU/mL [85]. Next, 40 μL of bacterial inocula were inoculated in 2 mL Eppendorf tubes containing 480 μL of TSB medium added with the same volume (480 μL) of different Zn and Fe nanoformulations. In the controls, the treatment was replaced with sterile distilled water. Treated cultures and control were incubated at 30 °C and 100 rpm for 48 h. Initial (T_0_) and final (T_1_) bacterial density were also estimated by plate count for each test tube in Tryptic Soy Agar (TSA) plates. For this purpose, 20 µL of each sample were uniformly spread in TSA medium and incubated at 30 °C for 24 h, serial dilutions (10^0^–10^−4^) in sterile distilled water were produced when needed to obtain between 30 and 300 colonies per plate [79]. 

Nanoformulations were also evaluated on the growth of soil fungal strains, *Fusarium oxysporum* and *Penicillium* sp., using the agar well diffusion method. Fungi were grown on Potato dextrose agar (PDA) medium at 25 °C for 10 days. Fungal disks were obtained using the back side of a sterile 200 μL micropipette tip. At the same time, fresh PDA culture plates were punched aseptically with a tip to make three 8mm wells. Wells were added with 100 µL of the nanoformulations at different concentrations (75, 250, and 500 ppm). A fungal disk was placed on the opposite side of the wells near the plate’s margin. Experimental controls were plates without NP addition; instead, the same volume of deionized water was added. Culture plates were incubated at 25 °C for 7 days. The zones of inhibition of fungal growth were measured [12,81,86].

#### 4.5.3. Impact on Early Plant Development in a Seedbed Assay

A 12-day seedbed experiment was carried out in a growth chamber to assess the early plant response to zinc and iron nanoformulations in radish (*Raphanus sativus*) and broccoli (*Brassica oleracea*). A completely randomized design was used. Twenty-four treatments were evaluated based on previous results of seed germination tests considering only the concentrations that exhibited beneficial effects; four formulations (bacteria-capped NPs, microalgae-capped NPs, uncapped NPs, mineral NP precursors) of two micronutrients (iron and zinc) at three concentrations (75, 250, 500 μg mL^−1^) were tested. Experimental controls were untreated plants irrigated with water. Three replicates (seedbed cavities) were considered per treatment, and eight plants per treatment were evaluated. Experiments were performed in plastic seedbeds (72 cavities; 5 × 5 × 6 cm) using sterile black soil Nutrigarden^®^ (Queretaro, Mexico). [84,87]. According to the manufacturer’s description and the regular black soil properties, this black-colored substrate is rich in clayey material with a neutral pH; it contains silica, calcium, carbonate, lime, potassium, aluminum, and magnesium [88]. 

Nanoparticles were tested in two fast-growing crops commonly grown as microgreens, radish (*Raphanus sativus* L. var. ‘Champion’) and broccoli (*Brassica oleracea* L. var. *italica*). For surface sterilization of the seeds, they were soaked in 70% ethanol for 30 s, followed by 5% sodium hypochlorite solution for 5 min, and five washes with sterile distilled water [89,90]. To induce germination, seeds were soaked in sterile distilled water with aeration overnight at room temperature. Ten seeds were sown per cavity at 0.5–1 cm depth. After emergence (emergence was considered when cotyledons were completely raised above the substrate), only eight healthy plants were maintained per pot, and selection was based on visual observations [91]. The growth chamber was maintained at 25 °C and 70% relative humidity under a 16 h:8 h light:dark cycle. The seedbeds were watered daily with tap water [82,87,92]. 

Treatments were applied three times during the test (days 1, 6, and 9), applying 2 mL of the formulations to the soil of the corresponding cavities of each treatment. After 12 days, experimental plants were harvested and evaluated for agronomic attributes [83]. Plant height (cm), root length (cm), leaf diameter (cm), and shoot fresh weight were measured, and data were averaged to analyze the results [90,93,94]. 

#### 4.5.4. Statistical Analysis

One-way analysis of variance ANOVA and Tukey Test (*p* < 0.05) were performed to analyze the experimental data using Jmp software version 17.0 (Jmp, Cary, NC, USA). Results were expressed as mean ± standard deviation (SD) considering three replicates [92,93,94].

## 5. Conclusions

This experimental work validated novel zinc and iron bio-nanofertilizers composed of ZnO- and γ-Fe_2_O_3_-NPs functionalized with a consortium of PGPR and a microalgae extract, covering the entire process from synthesis and characterization to the evaluation of its impact on soil microorganisms and the first stages of plant development. Considering all in vivo assays, treatments with bio-nanofertilizing potential were found for the different plant developmental stages; bacteria capped ZnO- and γ-Fe_2_O_3_-NPs at 75 ppm exerted significant improvements to seed germination and seedling vigor index, while ZnO- and γ-Fe_2_O_3_-NPs also treated with bacteria but applied at 250 ppm significantly improved the early plant development in a seedbed assay. Further experiments will be performed at later stages of plant development to determine their effects on plant productivity, biofortification, and stress responses. The study of new alternatives for nutrient management is of great relevance for modern agriculture to substitute conventional mineral fertilizers with efficient and safe nanoscale nutrients and improve crop productivity, plant nutritional value, and soil health. Products combining the beneficial effects of bio and nanofertilizers in a single preparation should be implemented in agriculture for sustainable food production. However, the risks of these novel bio-nanoformulations should be carefully studied, considering crop type, soil properties, and plant developmental stages for the safe use of nanomaterials in agriculture.

## Figures and Tables

**Table 1:** Zinc and iron nanoformulations for in vivo assays.

Assay	Objective	Treatments
Seed germination test	To determine if the biological agents used to formulate bio-nanofertilizers or the NP concentration significantly affect the germination of two important annual food-crops.	Twenty-four treatments corresponding to two micronutrients (iron and zinc), three capping conditions (uncapped or capped with bacteria (Bac) or microalgae (Alga)), and four concentrations (75, 250, 500, 1000 μg mL^−1^): Zn Bac 75, Zn Bac 250, Zn Bac 500, Zn Bac 1000, Zn Alga 75, Zn Alga 250, Zn Alga 500, Zn Alga 1000, Zn 75, Zn 250, Zn 500, Zn 1000, Fe Bac 75, Fe Bac 250, Fe Bac 500, Fe Bac 1000, Fe Alga 75, Fe Alga 250, Fe Alga 500, Fe Alga 1000, Fe 75, Fe 250, Fe 500, Fe 1000. Control (water-irrigated plants).
Soil microbial growth evaluation	To elucidate the nanotoxicity effects of different concentrations of nanoformulations on soil microorganisms.	Eighteen treatments resulting from two micronutrients (iron and zinc), three capping conditions (uncapped or capped with bacteria (Bac) or microalgae (Alga)) and three concentrations (75, 250, 500 μg mL^−1^): Zn Bac 75, Zn Bac 250, Zn Bac 500, Zn Alga 75, Zn Alga 250, Zn Alga 500, Zn 75, Zn 250, Zn 500, Fe Bac 75, Fe Bac 250, Fe Bac 500, Fe Alga 75, Fe Alga 250, Fe Alga 500, Fe 75, Fe 250, Fe 500. Control (water-irrigated plants).
Early plant response in a seedbed assay	To evaluate if the nanoscale of micronutrients, their concentration, and the use of capping agents influence the early plant response of two fast-growing plant species.	Twenty-four treatments corresponding to two micronutrients (iron and zinc), three capping conditions (uncapped or capped with bacteria (Bac) or microalgae (Alga)), two metal precursors (Prec), and three concentrations (75, 250, 500 μg mL^−1^): Zn Bac 75, Zn Bac 250, Zn Bac 500, Zn Alga 75, Zn Alga 250, Zn Alga 500, Zn 75, Zn 250, Zn 500, Zn Prec 75, Zn Prec 250, Zn Prec 500, Fe Bac 75, Fe Bac 250, Fe Bac 500, Fe Alga 75, Fe Alga 250, Fe Alga 500, Fe 75, Fe 250, Fe 500, Fe Prec 75, Fe Prec 250, Fe Prec 500. Control (water-irrigated plants).

**Table 2:** Bacterial growth at different concentrations of Fe and Zn nanoformulations.

Treatment	Initial Count T_0_ (CFU/mL)	Final Count T_1_ (CFU/mL)	% of Increase/Reduction of T_1_ with Respect to T_0_ *
Zn Bac 75	1.93 × 10^8^	2.63 × 10^8^	39.57 ± 4.53 bc
Zn Bac 250	2.76 × 10^8^	3.52 × 10^8^	26.88 ± 1.27 de
Zn Bac 500	1.85 × 10^8^	9.41 × 10^7^	−49.57 ± 0.79 k
Zn Alga 75	5.38 × 10^7^	7.15 × 10^7^	32.90 ± 0.14 cd
Zn Alga 250	5.49 × 10^7^	6.75 × 10^7^	23.01 ± 0.08 e
Zn Alga 500	5.39 × 10^7^	3.70 × 10^7^	−31.05 ± 0.43 ij
Zn 75	3.76 × 10^7^	8.41 × 10^7^	123.87 ± 0.05 a
Zn 250	1.24 × 10^8^	1.73 × 10^8^	41.13 ± 2.06 b
Zn 500	4.37 × 10^7^	2.15 × 10^7^	−51.25 ± 0.56 k
Fe Bac 75	2.01 × 10^8^	2.75 × 10^8^	36.10 ± 1.04 bc
Fe Bac 250	2.18 × 10^8^	2.66 × 10^8^	21.36 ± 1.34 e
Fe Bac 500	2.09 × 10^8^	2.03 × 10^8^	−3.32 ± 0.30 g
Fe Alga 75	1.54 × 10^8^	2.00 × 10^8^	30.32 ± 0.04 d
Fe Alga 250	1.48 × 10^8^	9.87 × 10^7^	−34.07 ± 1.04 j
Fe Alga 500	1.49 × 10^8^	7.11 × 10^7^	−52.73 ± 0.87 k
Fe 75	1.50 × 10^7^	1.66 × 10^7^	10.46 ± 0.18 f
Fe 250	2.90 × 10^7^	2.13 × 10^7^	−28.08 ± 2.36 hi
Fe 500	1.34 × 10^7^	1.01 × 10^7^	−25.55 ± 1.33 h
Control	6.21 × 10^7^	6.85 × 10^7^	9.95 ± 0.51 f

* Green and red color indicate increase and reduction, respectively. Results are presented as mean ± standard deviation, and letters denote significant statistical differences between treatments (*p* < 0.05).

## Data Availability

Not applicable.
